# Effects of Fast-Tempo and Binaural Beat Therapy Music during Warm-Up on Repeated Sprint Ability Test Performance among Young Soccer Players

**DOI:** 10.3390/brainsci14070673

**Published:** 2024-07-01

**Authors:** Songyan Wang, Cheng Liu, Lin Zhang, Cheuk-Kwan Sun, Shang-Yu Yang

**Affiliations:** 1 Football Academy, Wuhan Sports University, Wuhan 430079, China; 2003003@whsu.edu.cn; 2 Department of Physical Education, Huazhong University of Science and Technology, Wuhan 430074, China; fatimaliu@hust.edu.cn (C.L.); zhanglin@hust.edu.cn (L.Z.); 3 Department of Emergency Medicine, E-Da Dachang Hospital, I-Shou University, Kaohsiung 80785, Taiwan; 4 School of Medicine for International Students, College of Medicine, I-Shou University, Kaohsiung 80785, Taiwan; 5 Department of Healthcare Administration, Asia University, Taichung 41354, Taiwan

**Keywords:** soccer, binaural beat therapy, music therapy, repeated-sprint ability test, athletes

## Abstract

This prospective crossover study aimed to investigate the effect of binaural beat therapy music on soccer player performance. Between July 2023 and December 2023, 45 athletes (31 females/14 males, mean age = 20.47 ± 0.99) wore Bluetooth earphones through which one of the following was given during initial 20 min warm-up exercises before undergoing repeated sprint ability tests: no music/fast-tempo music/fast-tempo music with background binaural beat therapy music. Heart rate change after warm-up exercises/repeated sprint ability (RSA) tests and the time to finish RSA tests were recorded. Despite no significant difference in heart rate increase after warm-up between the two genders regardless of intervention, larger increases after RSA tests were found in males following any of the three interventions (all *p* < 0.01) with the most notable difference observed after fast-tempo music (*p* < 0.0001). A significant effect size (r = 0.2) correlated with fast-tempo music during warm-up in either gender. Binaural beat therapy music during warm-up reached a significant effect size only when all participants were considered, suggesting limited benefits.

## 1. Introduction

Soccer is a team-based high-intensity sport that involves extensive running and sprinting for a long duration mingled with active and passive recovery periods [1,2]. Previous studies have shown that a soccer player can cover a distance of 9–14 km, a high-speed distance of 0.7–3.9 km, and a sprint distance of 0.2–0.6 km in a single match that entails up to 600 accelerations [3,4]. Warm-up exercises, which typically include short-duration but high-intensity activities, have been reported to promote athletes’ excitability through increasing intramuscular temperature, nerve conduction rate, and metabolic reactions [5]. Additionally, warm-up exercises may boost self-confidence and the ability to focus by mentally preparing an athlete for the upcoming physical challenge [6]. A systematic review focusing on adult soccer players has demonstrated an improvement in muscle strength by 3.5–4.2% as well as speed and jumping by 1–20% after warm-up exercises [5]. The repeated sprint ability (RSA) test, which involves six 40 m sprints with 180° direction change interspersed with a 20 s passive recovery period [7], has been shown to correlate with the running distances covered during a match among top-level professional soccer players [7]. Prior studies have also reported the ability of the test to distinguish between professional and amateur soccer players [8,9]. Physiologically, RSA represents neuromuscular and metabolic abilities as reflected by maximal sprint speed and phosphocreatine (PCr) recovery rate, respectively [10,11].

In addition, prior investigations have demonstrated the role of music, especially fast-paced melodies, in boosting arousal in various sports [12,13]. Tempo, the speed at which a passage of music is played, is usually measured by beats per minute (bpm). The three common music tempos are slow (60–108 bpm), moderate (108–120 bpm), and fast (120–168 bpm) [14]. A previous study using repeated sprint tests has reported that listening to fast-tempo music (>130–140 bpm) when engaging in warm-up exercises could enhance the arousal of athletes [13] and their sport performance compared to those who did not listen to music during the warm-up period [15]. Fast-tempo music is known to increase heart rate, which is one of the purposes of warm-up exercises [16]. Consistently, a previous investigation targeting volleyball players who listened to fast-tempo music (>140 bpm) during a 10 min session of warm-up exercises has shown a higher heart rate compared to non-listeners [17]. Similar findings have also been reported in basketball players who exhibited a higher heart rate both after a 20 min warm-up period and at 10 min after starting the game if they listened to fast rhythm music during their warm-up exercises compared to those who listened to slow rhythm music, highlighting the beneficial effect of fast rhythm music on their sport performance [18].

The performance of soccer players is adversely affected by a number of factors, such as inattention [19]. Binaural beat therapy (BBT), which is based on alerting the brain to the difference in frequency (i.e., 40 Hz), involves giving an individual auditory signal of slightly different frequencies (e.g., 300 and 340 Hz) on each ear [20]. While the low frequency sound (e.g., delta) in BBT is related to relaxation [21], the high frequency sound (e.g., gamma) is linked to attention and alertness [22]. This is consistent with the previous finding of attention reinforcement through high frequency neurofeedback of the frontal cortex [23]. Additionally, previous studies have shown that BBT with a gamma frequency could promote attention [24], relieve anxiety [25], and alleviate negative emotions [26]. Brain waves reflect rhythmic patterns of neuronal activity as a result of synchronized electrochemical pulses from different groups of neurons in the central nervous system (CNS) [27]. Gamma brainwaves at frequencies of about 40 Hz and above are associated with the states of attention and focus governed by the prefrontal cortex [28], which is also involved in the modulation of affective, motivational, and performance processes in sports and exercise [28]. Nevertheless, despite the reported benefits of BBT, its role in enhancing repeated sprint ability in soccer players remains unclear.

In addition, although fast-tempo music has been found to reinforce the arousal and physical performance of athletes participating in different sport activities [13,15,17,18], whether its combination with BBT could provide an additional or even synergistic effect has not been investigated. Therefore, taking into consideration the reported positive impact of fast-tempo music [18] and the potential beneficial effect of BBT music on sport/exercise performance [29], we combined fast-tempo with BBT music to elucidate the influence of listening to BBT music during warm-up exercises and the RSA test in young soccer players.

## 2. Materials and Methods

### 2.1. Study Design and Procedure

**Figure 1:**
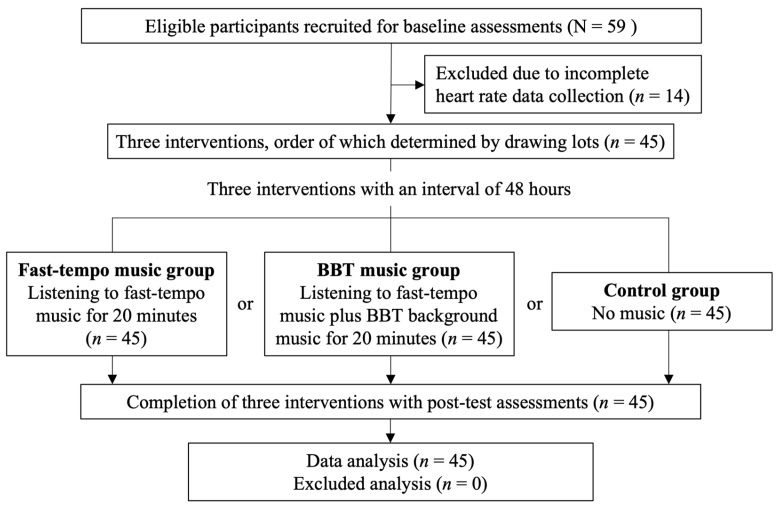
Study protocol and interventions. BBT: binaural beat therapy.

This is a prospective study with a quasi-experimental design. Between 30 July 2023 and 30 December 2023, student volunteers of a national soccer academy were recruited after a full explanation of the procedures of the current study. All participants were required to submit information about their age, gender, body mass index, seniority in university, playing position, experience as a player, competitive level, as well as their habits of cigarette smoking and alcohol consumption in a questionnaire. Each participant received three interventions (i.e., no music, fast tempo music, and BBT music) during a 20 min warm-up session in random order with an interval of 48 h between the interventions (Figure 1). All participants underwent a repeated sprint ability (RSA) test immediately after starting the warm-up period.

### 2.2. Participants

Using the G*Power 3.1 software (Wilcoxon signed-rank test) with an effect size, alpha level, and power being set at 0.5, 0.05, and 0.9, respectively, the minimal number of participants required to produce statistically significant results was 38. The inclusion criteria were (1) adults of age 18 or above regardless of gender, and (2) students devoted to soccer training at a university with prior participation in at least one national tournament. On the other hand, those who (1) had hearing impairment, (2) were left-handed (because of concern about right hemisphere dominance), (3) experienced musculoskeletal injuries in the past month, or (4) met the criteria of habitual smokers (defined as smoking at least one cigarette a day for at least six months) or drinkers (defined as daily alcohol consumption regardless of quantity for over six months) were excluded. The protocol and procedures of the current study were reviewed and approved by the Institutional Review Board of Wuhan Sports University (IRB no.: 202-30-80). All participants signed an informed consent before participation. The whole study was conducted in compliance with the Declaration of Helsinki.

### 2.3. Study Parameters

For the current study, we adopted a warm-up process modified from the F-MARC 11+ program developed by the International Federation of Association Football medical research center (F-MARC) [30]. A systematic review showed that dynamic stretching and the F-MARC 11+ program could enhance subsequent performance in soccer players [5]. Accordingly, the modified 20 min warm-up process in the present study comprised forward jogging (3 min), sideways jumping jacks and lunges (i.e., dynamic stretching) (5 min), two-player ball-passing (5 min), and two-group confrontation (soccer passing drills) (7 min).

RSA test has been widely utilized in the assessment of sport performance among soccer players [31] because of similar metabolic and cardiopulmonary responses generated through RSA test to those that the players actually experience in a soccer game [2]. The RSA test, which was conducted on a synthetic soccer field, comprises six 40 m shuttle sprints (20 plus 20 m) interspersed with 20 s of passive recovery [7]. The participants started from a line located 50 cm behind an infra-red segmented timer, sprinted for 20 m, and touched a line with one foot before returning to the starting line as soon as possible. Following 20 s of passive recovery, the participant repeated the whole process. Five seconds before starting each sprint, the participants assumed the ready position before the start signal. Participants with a total duration of the RSA test over 2 min were excluded from the present study. During the periods of warm-up and repeated sprint ability (RSA) test, the heart rate of each participant was digitally recorded (Polar H1 heart rate monitor, Polar Electro, Kempele, Finland). An infra-red segmented timer (patent no.: CN203746222U, Wuhan, China) was used to automatically record the time taken for the participants to complete their sprints with a shorter time representing better performance. All data were transferred to a computer for storage and analysis.

### 2.4. Interventions

To avoid interfering with the actual performance in the RSA test, all interventions were given to the participants only during the 20 min warm-up period. The warm-up process was overseen by the first author (SW) who is an associate professor who possesses a Chinese Football Association match supervision license and participated in soccer-related research, while evaluation and data collection were conducted by the second author (CL) who is a PhD in healthcare administration and is currently a lecturer in exercise physiology with experience in physical-education-related data collection and analysis. The current study adopted a crossover design with each individual participating in all three interventions (i.e., no music, fast tempo music, and fast-tempo music plus BBT music) in a random order (https://www.randomizer.org/, accessed on 30 July 2023). An interval of 48 h was required between the interventions. Before the warm-up procedures, all participants were required to wear Bluetooth earphones through which the chosen intervention (i.e., fast-tempo music with or without BBT music) was delivered at a self-selected volume taking into account individual differences in auditory perception and variation in environment auditory interference. All participants were instructed to adjust the volume of music to a level at which they felt comfortable. All interventions began immediately after the start of the warm-up exercises and lasted for 20 min until the end of the warm-up process. The participants were randomly assigned to three groups (Figure 1): (1) Fast-tempo music group: Fast-paced music (>130 bpm) was delivered through the earphones as previously described [32]. Six rhythms, which were shown to enhance exercise performance in sprinters, namely “Cigarette Daydreams”, “Hero”, “Shanghai Alice Magic Orchestra”, “Toy War”, “Where to Jun”, and “Dream Land Days” [33], were chosen. (2) Binaural beat therapy (BBT) music group: In addition to fast-paced music (>130 bpm), background BBT music comprising beat and gamma waves of slightly different frequencies between the two sides (i.e., beta: left ear 380 Hz, right ear 350 Hz; gamma: left ear 390 Hz, right ear 350 Hz) was delivered through Bluetooth earphones to the participants for durations of 5 and 15 min, respectively. Beta and gamma waves tend to dominate the frontal region (Fp1, Fp2) and somatosensory area (C3, C4) which are related to the development of psychomotor skills [34]. (3) Control group: Participants wore their earphones during the entire 20 min warm-up duration but no sound signal was sent. Both fast-tempo and BBT music pieces were compiled with Adobe Audition CC 2021 software.

Taking into consideration the potential confounding endocrine effects of circadian rhythm, all studies were conducted during the same period in the morning (i.e., 9:00–11:00 a.m.) as previously described [35]. The location of warm-up exercises and RSA test was an open soccer field with an artificial lawn of size 105 m × 68 m with temperature and humidity at 25 ± 5 °C and 50 ± 5%, respectively. One day before testing, all participants received a message through their mobile phones containing the following information: (1) maintenance of ordinary daily diet and no alcohol consumption within 24 h before testing; (2) no vigorous exercise within 24 h prior to testing; (3) keeping normal sleep schedule; (4) wearing the same sports shoes in different test sessions; (5) avoidance of excessive eating and water intake before testing. On the day of the test, all participants completed a questionnaire to ensure their compliance with the instructions. The second author (CL) examined the questionnaire as well as confirmed the correct wearing and function of the instruments (i.e., Bluetooth earphones and heart rate monitor).

### 2.5. Statistical Analysis

Descriptive statistics were applied where appropriate. Average values were shown as medians with interquartile ranges. For continuous variables (i.e., age, body mass index, or mean heart rate during warm-up and RSA test) that were not normally distributed (i.e., Shapiro–Wilk test, *p* < 0.05), Mann–Whitney U test was adopted to assess the significance of difference between female and male participants. Mann–Whitney U test was also used to determine the significance of change in heart rate before and after warm-up exercise as well as that before and after RSA test. Chi-squared test was utilized to evaluate the difference in seniority in university, playing position, experience as a player, and competitive level between the female and male groups. Wilcoxon signed-rank test was used to determine the significance of difference in median heart rate during the warm-up period and RSA test as well as time taken for the RSA test. Effect sizes acquired using Cohen’s r were used to assess the standardized mean difference between two interventions (i.e., no music vs. fast-tempo music, no music vs. fast-tempo plus BBT background music, and fast-tempo music vs. fast-tempo plus BBT background music). Effect sizes corresponding to Cohen’s r values of 0.2, 0.5, and 0.8 are regarded as small, medium, and large [36]. Statistical analyses were performed with SPSS 25 for Mac (IBM Corp., Armonk, NY, USA). A probability value, *p*, less than 0.05 was considered statistically significant.

## 3. Results

### 3.1. Characteristics of the Participants

Of the 59 volunteers initially recruited for the current study, 14 were excluded because of incomplete heart rate data collection (Figure 1). Finally, 14 males and 31 females with a mean age of 20.47 ± 0.99 and a mean body mass index of 21.03 ± 2.28 were recruited for the current study. The demographic characteristics, seniority in university, playing position, experience as a player, and competitive level are shown in Table 1. Male participants were older, had a higher body mass index, and were more senior in university than their female counterparts (all *p* < 0.001). On the other hand, the competitive level was significantly higher in females than that in males (*p* = 0.037). No difference in playing position and experience as a player was noted between the two groups (Table 1).

### 3.2. Gender Difference in Heart Rate Increase after Warm-Up Exercise and RSA Test

**Figure 2:**
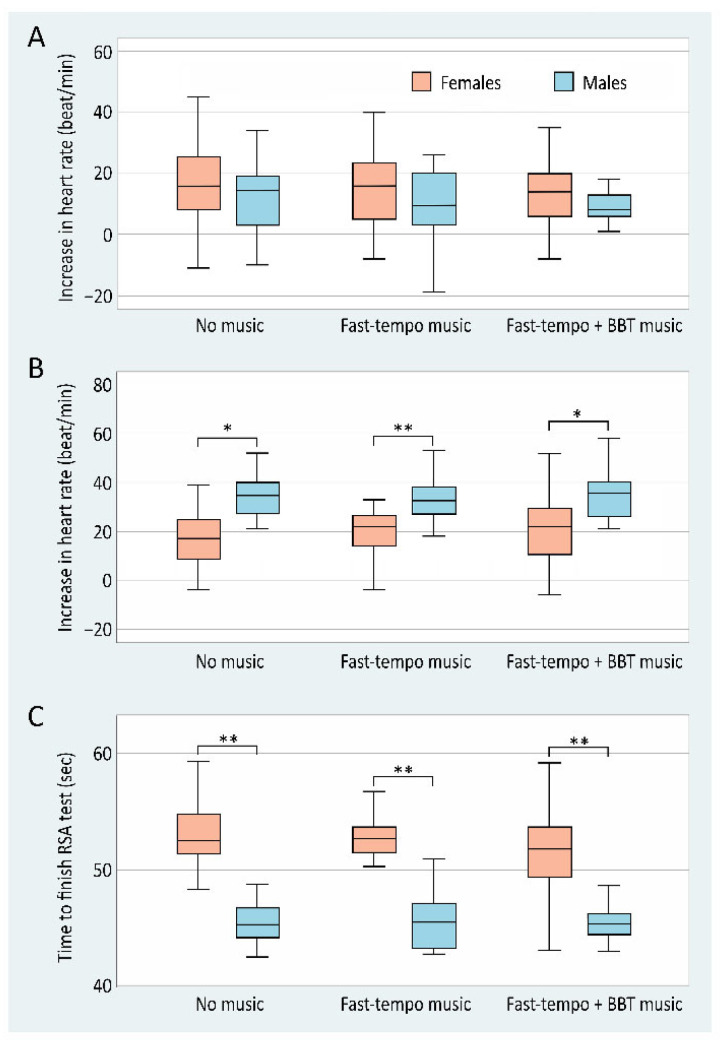
Comparisons of changes in heart rate during (**A**) warm-up exercises and (**B**) repeated sprint ability (RSA) test, as well as (**C**) time to finish RSA test between female (*n* = 31) and male (*n* = 14) participants. Significance of difference determined by Mann–Whitney U test; * *p* < 0.005, ** *p* < 0.0001.

The increase in heart rate after warm-up exercises was not significantly different between female and male participants regardless of the type of music given to them through their earphones (i.e., no music, fast-tempo music with or without BBT background) (Figure 2A). On the other hand, the increase in heart rate was significantly larger in males than that in females after the RSA test in all three conditions (all *p* < 0.01), especially when listening to fast-tempo music with (*p* = 0.005) or without (*p* < 0.0001) a BBT background compared with the no music group (*p* = 0.003) (Figure 2B).

### 3.3. Gender Difference in RSA Test Performance

The time for completing the RSA test was significantly shorter (i.e., better performance) in male participants than that in their female counterparts regardless of the type of music that they listened to (i.e., no music, fast-tempo music with or without a BBT background) (all *p* < 0.0001) (Figure 2C).

### 3.4. Effect Sizes of Three Interventions on Changes in Heart Rate and Time for Completing RSA Test

To assess the impact of the three interventions on heart rate during warm-up exercises and RSA tests as well as that on the time to complete the RSA test, we used the Wilcoxon signed-rank test to compute the effect size (Table 2). The results showed significant effect sizes in the increase in heart rate after RSA tests in both females (r = 0.2) and males (r = 0.2) when they listened to fast-tempo music compared to the same occasion when they were given no music through their earphones. When all participants were considered, the effect size remained significant for heart rate after the RSA test when comparing between the fast-tempo music and no music conditions. In addition, the effect size of BBT plus fast-tempo music was significant (r = 0.22) compared to the situation when no music was delivered to the participants. However, no significant differences in RSA test performance (i.e., time to finish the test) were noted between the three interventions in both female and male participants (Table 2).

## 4. Discussion

Despite previous investigations into the correlation between fast-tempo music and heart rate in exercise [37,38,39], the current study was the first to combine BBT background music with fast-tempo music under two exercise conditions (i.e., warm-up and the RSA test) and to compare the effect of gender on the outcomes. Although no significant difference was noted in the increase in heart rate between the two genders after warm-up exercises regardless of the intervention, our analysis showed a more significant increase in heart rate in male participants during the RSA test than their female counterparts, especially when they listened to fast-tempo music. For both genders, the increase in heart rate during the RSA test reached a significant effect size when the participants listened to fast-tempo music compared to the situation in which no music was delivered through their earphones. In contrast, the effect size of a change in heart rate between the conditions of fast-tempo plus BBT background music and no music was significant only when all of the participants were considered, regardless of gender. Therefore, our results highlighted the significance of fast-tempo music in enhancing heart rate during the RSA test, which is more strenuous physical exertion than warm-up exercises, in both genders. On the other hand, the addition of BBT background music offered neither an additional nor a synergistic effect in this setting. The current study also demonstrated a higher increase in heart rate during the RSA test as well as a shorter time required to complete the test in male subjects compared to female participants, suggesting the ability to work at a higher intensity in the former. Our finding was compatible with that reported in a comprehensive review that underscored the impact of biological sex on physical performance [40]. That study reported that adult males are typically stronger and faster than their age-matched female counterparts despite a similar training status, with the former typically outperforming the latter by 10–30% [40]. Focusing on the time to complete an RSA test in the control groups (i.e., those without music intervention), our study showed that males outperformed females by 16.3%, which was compatible with the finding of that review. Nevertheless, our results showed no significant impact of any of the three interventions on RSA test performance in both genders. Previous studies have reported sex differences in emotional and electrophysiological responses [41] as well as physical reactions [42] to music. Not only were women shown to be more electrophysiologically sensitive to arousing and unpleasant music compared with men [41], but they also responded more favorably to self-selected music in terms of enhancing motivation to exercise than their male counterparts [42]. On the other hand, no music-related gender difference in emotional response [41] and anaerobic performance [42] was noted.

Music is thought to enhance athletic performance through several mechanisms, including elevation of the level of arousal, suppression of the sensation of fatigue, reinforcement of motor coordination and synchronization, as well as the promotion of relaxation [43]. When comparing between the two genders, no significant difference in heart rate was noted between the two genders after warm-up exercises. In contrast, the present study showed a more significant elevation in heart rate in males than in females after the RSA test, with the former completing the test significantly faster than the latter regardless of the interventions. Therefore, the results implied a higher readiness or explosiveness in male than in female participants when subjected to strenuous exercises. As RSA test performance is known to be an indicator of maximal aerobic capacity [44], the significant increase in heart rate after listening to fast-tempo music in participants of both genders may suggest an enhancement of readiness for oxygen-demanding physical activities but not for less demanding warm-up exercises. Consistently, previous studies have shown that although warm-up exercise does not increase oxygen consumption [45,46], it may allow subsequent tasks to begin with an elevated baseline oxygen consumption so that less of the initial work will be completed anaerobically, with more anaerobic capacity being reserved for the later task [16]. This hypothesis is endorsed by previous investigations that demonstrated a greater aerobic contribution [47,48,49,50] and/or a reduced oxygen deficit [49,51,52,53] when physical exertions are preceded by warm-up exercise.

The current study also investigated the potential benefits of adding BBT music as background music to fast-tempo music in this setting. The rationale for utilizing BBT background music for athletic training is based on the finding of an improvement in concentration among children and adolescents with attention-deficit/hyperactivity disorder through BBT–music-elicited enhancement of their gamma brain wave activity [24]. A previous study has shown the positive role of concentration in helping athletes make split-second decisions under the pressures of competition, thereby improving their performance [54]. On the other hand, although our findings demonstrated a significant effect size (r = 0.22) regarding the impact of BBT background music on heart rate after the RSA test when all participants were considered, the effect size became non-significant when those of each gender were analyzed separately (Table 2). Hence, compared with fast-tempo music, our results implied limited benefits of adding BBT background music to fast-tempo music during the warm-up period in preparing the participants for the RSA test. Our findings were inconsistent with those of a previous study that demonstrated a reinforcement of anaerobic exercise capacity among elite adolescent volleyball players after listening to BBT music during the warm-up period [17]. One possible explanation may be a relatively low demand for concentration and precision in the RSA test that mainly involves short distance sprints with direction change in comparison with more concentration-demanding sports such as archery, gymnastics, badminton, table tennis, and volleyball. Further studies focusing on the possible beneficial effects of BBT music on these sports are warranted to elucidate this issue.

### Strengths and Limitations

Previous reports on the effects of BBT music on athletes mostly focused on the relaxing property of BBT music on positive thinking [18] and sleep quality using the alpha frequency. The current study was the first to investigate the effect of BBT music at the gamma frequency on athletic performance as well as the first to combine BBT and fast-tempo music. Additionally, compared with previous studies [18,55], our investigation had a relatively large sample size that enabled a comparison of sex differences in response to music interventions. Nevertheless, there were several limitations in the current study. First, despite our relatively large sample size, it was still too small to reach a robust conclusion. Additionally, a female predominance in our recruited subjects may contribute to the lack of significance in RSA test performance between the three groups. Therefore, despite the sample size being over the minimal number of participants required to determine statistical significance according to G*Power, further large-scale studies are warranted to verify our findings. Second, to avoid the participants carrying more sophisticated equipment on their bodies that may impede their normal physical performance, only their heart rates and time to finish the RSA test were recorded, so that information about changes in other physiological parameters (e.g., arterial oxygen saturation or oxygen consumption) was not available for comparison. Hence, whether our finding of an increase in heart rate in response to fast-tempo music reflected an enhanced readiness for oxygen-consuming activities remains unclear. Third, subjective ratings of exertion and psychological states among the participants that may affect their physical performance were not investigated. Fourth, individual differences in physical performance may bias our findings. Nevertheless, we selected those who attended at least one national tournament to ensure their meeting a fair athletic standard to minimize possible bias arising from discrepancies in physical status. Fifth, although the current study found no gender difference in response to music interventions, endocrinological changes (e.g., cortisol level) were not checked to validate our findings. Sixth, because the present investigation only focused on young athletes, our results may not be extrapolated to other populations such as non-athletes and various age groups. Finally, only the participants’ performance on the RSA test was assessed without including exercises that require a higher level of concentration and precision, especially those involving making split-second decisions (e.g., penalty kicks), so that the benefits of BBT music may be obscured. Further studies focusing on the exploration of the psychological impact of BBT interventions alongside their physiological effects based on more comprehensive parameters in different populations are needed to uncover complex interactions that the current study failed to address.

## 5. Conclusions

The results of the current study indicated a gender difference in repeated sprint ability (RSA) test performance, with male participants showing a significantly larger increase in heart rate during the test and shorter time to finish the test than their female counterparts. Such an increase in heart rate following RSA test reached significant effect sizes in both genders following their listening to fast-tempo music during their warm-up period. However, the addition of binaural beat therapy (BBT) background music was associated with a significant effect size only when all participants were considered, indicating its limited benefit in this physical training setting. Further studies are warranted to elucidate the possible beneficial effect of BBT music in other concentration- or precision-demanding sports.

## Figures and Tables

**Table 1:** Characteristics of the participants (*n* = 45).

	Total	Female (*n* = 31, 68.9%)	Male (*n* = 14, 31.1%)	*t*/*Z*	*p*
Age [Median (IQR)] ^a^	21.0 (20.0, 21.0)	20.0 (19.0, 21.0)	21.0 (21.0, 21.0)	−3.433	0.001
BMI [Median (IQR)] ^a^	20.2 (19.5, 22.6)	19.8 (19.0, 20.6)	23.0 (22.4, 23.6)	−6.602	<0.0001
Seniority in university (*n*, %) ^b^				19.439	<0.0001
1st year	9 (20.0%)	9 (29.0%)	0		
2nd year	13 (28.9%)	13 (41.9%)	0		
3rd year	23 (51.1%)	9 (29.0%)	14 (100%)		
Playing positions (*n*, %) ^b^				2.140	0.830
Goalkeeper	2 (4.4)	2 (6.5)	0		
Full back	8 (17.8)	5 (16.1)	3 (21.4)		
Center back	2 (4.4)	2 (6.5)	0		
Central midfield	12 (26.7)	8 (25.8)	4 (28.6)		
Forward	8 (17.8)	5 (16.1)	3 (21.4)		
Side MidFielder	13 (28.9)	9 (29.0)	4 (28.6)		
Experience as players (*n*, %) ^b^				3.280	0.350
1–2 year	1 (2.2)	1 (3.2%)	0		
3–4 year	1 (2.2)	0	1 (7.1)		
5–6 year	6 (13.3)	5 (16.1)	1 (7.1)		
Over 7 years	37 (82.2)	25 (80.6)	12 (85.7)		
Competitive level (*n*, %) ^b^				4.360	0.037
Higher division	20 (44.4%)	17 (54.8%)	3 (21.4%)		
Middle division	25 (55.6%)	14 (45.2%)	11 (78.6%)		

Significance of difference determined with ^a^ Mann–Whitney U test and ^b^ Chi-squared test; BMI: body mass index; IQR: interquartile range.

**Table 2:** Effect size (r) of Wilcoxon signed-rank test (*n* = 45).

Gender			No Music vs. Fast-Tempo Music			Fast-Tempo Music vs. BBT Plus Fast-Tempo Music			No Music vs. BBT Plus Fast-Tempo Music		
			Z	*p*	Effect Size (r)	Z	*p*	Effect Size (r)	Z	*p*	Effect Size (r)
All	Heart rate during warm-up		−1.17	0.24	0.17	−0.84	0.40	0.13	−0.01	1.00	0.00
	RSA test	Heart rate	−1.80	0.07	0.27 *	−0.01	0.99	0.00	−1.47	0.14	0.22 *
		Time (s)	−0.27	0.79	0.04	−1.28	0.20	0.19	−0.88	0.38	0.13
Female	Heart rate during warm-up		−1.68	0.09	−0.25	−0.30	0.77	0.04	−1.29	0.20	−0.19
	RSA test	Heart rate	−1.31	0.19	0.20 *	−0.23	0.82	0.03	−1.19	0.23	0.18
		Time (s)	−0.06	0.95	0.01	−1.12	0.26	0.17	−1.08	0.28	0.16
Male	Heart rate during warm-up		-0.22	0.83	0.03	−0.47	0.64	0.07	−0.79	0.43	0.12
	RSA test	Heart rate	−1.34	0.18	0.20 *	−0.45	0.66	0.07	−0.77	0.44	0.11
		Time (s)	−0.5	0.57	0.08	−0.53	0.59	0.08	−0.09	0.93	0.01

* Significant effect size; BBT: binaural beat therapy music; RSA: repeated sprint ability.

## Data Availability

The data that support the findings of this study are available on request from the corresponding author. The data are not publicly available due to privacy or ethical restrictions.
